# Evaluation of *in vitro* neuronal platforms as surrogates for *in vivo* whole brain systems

**DOI:** 10.1038/s41598-018-28950-5

**Published:** 2018-07-17

**Authors:** Anna M. Belle, Heather A. Enright, Ana Paula Sales, Kristen Kulp, Joanne Osburn, Edward A. Kuhn, Nicholas O. Fischer, Elizabeth K. Wheeler

**Affiliations:** 1 Engineering Directorate, Lawrence Livermore National Laboratory, Livermore, California, USA; 2 Physical and Life Sciences Directorate, Lawrence Livermore National Laboratory, Livermore, California, USA

## Abstract

Quantitatively benchmarking similarities and differences between the *in vivo* central nervous system and *in vitro* neuronal cultures can qualify discrepancies in functional responses and establish the utility of *in vitro* platforms. In this work, extracellular electrophysiology responses of cortical neurons in awake, freely-moving animals were compared to *in vitro* cultures of dissociated cortical neurons. After exposure to two well-characterized drugs, atropine and ketamine, a number of key points were observed: (1) significant differences in spontaneous firing activity for *in vivo* and *in vitro* systems, (2) similar response trends in single-unit spiking activity after exposure to atropine, and (3) greater sensitivity to the effects of ketamine *in vitro*. While *in vitro* cultures of dissociated cortical neurons may be appropriate for many types of pharmacological studies, we demonstrate that for some drugs, such as ketamine, this system may not fully capture the responses observed *in vivo*. Understanding the functionality associated with neuronal cultures will enhance the relevance of electrophysiology data sets and more accurately frame their conclusions. Comparing *in vivo* and *in vitro* rodent systems will provide the critical framework necessary for developing and interpreting *in vitro* systems using human cells that strive to more closely recapitulate human *in vivo* function and response.

## Introduction

Efforts to develop human cell-based microphysiological systems that recapitulate organ function are aimed at reducing the extensive use of experimental animal models, which often inaccurately predict human specific responses^1,2^. Fully validated systems hold promise for evaluating new drugs, characterizing toxicants, and aiding in the elucidation of disease mechanisms. A system that faithfully captures important brain function would be particularly relevant; neurological diseases and injuries are significant causes of morbidity and mortality but are often intractable to development of new and effective therapeutics when relying solely on animal models.

Neuronal cultures are frequently used to evaluate the effects of drugs or toxicants on cellular responses, taking advantage of multi-electrode arrays (MEAs) to provide a means to non-invasively interrogate neuronal cell health and function^3–7^. However, little to no information is available on the extent to which these *in vitro* systems reflect what is observed *in vivo*, despite calls from the research community to examine this relationship more closely^4,8^. While organotypic brain slices that preserve cellular architecture and circuitry are often superior to dissociated neurons as *in vitro* models, cell cultures are widely used as diagnostic screening platforms^9,10^. Furthermore, neuronal cultures, in contrast to brain slices cultured on MEAs, are more readily adapted to human-relevant systems by utilizing either human primary or stem-cell derived neurons. As such, a direct comparison of dissociated neuronal cultures to whole brain provides important and necessary information to frame the relevance of *in vitro* data sets. To that end, we chose to compare the prefrontal cortex *in vivo* to dissociated cortical neurons *in vitro*. The prefrontal cortex was chosen due to ease of reproducibly recording single-unit activity from neuronal probes in this brain region *in vivo* and its overall relevance in evaluating neuronal responses to chemical exposure^11–14^. The dissociated cortical neuron culture contains the representative neuronal cell types found in the prefrontal cortex^15^.

While the cellular milieu and network inputs differ significantly between neurons in an isolated culture compared to neurons in their native brain environment, many responses, particularly response trends, are expected to be similar. To verify these expectations, however, a systematic and quantitative comparison of functional responses to chemical stimuli between these systems is necessary. Herein, both experimental systems were exposed to two different drugs known to induce dual responses (i.e. both increases and decreases in single neuron firing rates): atropine and ketamine. These well-characterized drugs elicit effects both *in vivo* and *in vitro* and are known to work through interaction with different receptor populations involved in cortical function. Atropine is a muscarinic receptor antagonist affecting both the central and parasympathetic nervous systems^16^. Muscarinic receptors regulate acetylcholine release and neuronal spiking activity and are located on both excitatory and inhibitory cortical neurons^17^. Ketamine is an N-methyl-D-aspartate (NMDA) receptor antagonist that blocks receptor activity. It can elicit an increase or decrease in firing rate depending on the dosage^18–20^, cell type^21^, cell state^12^ and through modulation of other glutamatergic receptors^21^. One important consideration when directly comparing *in vitro* to *in vivo* responses is the possible effect of metabolism or modification of the parental drug molecule *in vivo* (which would not be similarly metabolized in a simple *in vitro* system). While no neuroactive metabolites have been reported for atropine, metabolites for ketamine (i.e. (R,S)-norketamine or (2R,6R)-hydroxynorketamine) have been shown to elicit neuronal responses^22,23^. These secondary effects may influence the overall outcome in an *in vivo* system. In this study, the administered doses of the drugs were normalized using reported region-specific brain accumulation values for the parent compound to allow for direct comparisons between the systems^22,23^.

Conducting direct comparisons between *in vivo* and *in vitro* experimental systems provides a more informed framework in which to interpret functional data and responses to chemical stimuli and has important implications for increasing the significance of drug development and toxicology studies. Our study illustrates that the relevance of results obtained from *in vitro* cultures likely depends on many factors including indirect neuronal effects that may be a result of neuroactive metabolites or the interaction of the parent compound with non-neuronal targets.

## Results

### Spontaneous single-unit activity

**Figure 1:**
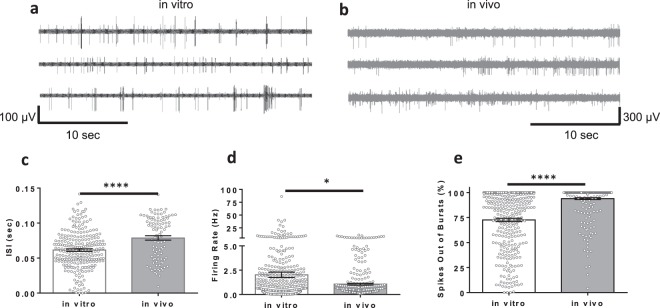
Baseline activity of neurons recorded from *in vivo* and *in vitro* multielectrode arrays (MEAs). Three representative electrodes with neuronal activity are shown for *in vitro* and *in vivo* recordings in (**a**,**b**), respectively. Comparisons of firing features are shown including: (**c**), interspike interval (ISI) (0.061 ± 0.001 sec *in vitro* [n = 247] and 0.079 ± 0.002 sec *in vivo* [n = 96]), (**d)**, firing rate (2.0 ± 0.2 Hz *in vitro* [n = 474]) and 1.06 ± 0.09 Hz *in vivo* [n = 362] and (**e)**, percent of spikes out of bursts (73 ± 1% *in vitro* [n = 322] and 93.9 ± 0.9% *in vivo* [n = 345]). Data are shown as mean ± s.e.m. Asterisks (*) indicate significance in Mann-Whitney U test. *p < 0.01, ***p < 0.001.

Neuronal electrophysiology was compared between rodent cortical neurons cultured on planar MEAs (*in vitro*) and awake, freely moving rats using implantable MEA probes (*in vivo*). Key similarities and differences for single-unit activity between these two neuronal systems were assessed by comparing both baseline electrophysiological activity and response to two classes of well-characterized neuroactive chemicals. Spontaneous (baseline) single-unit extracellular neuronal spiking activity in both *in vitro* and *in vivo* systems was compared by quantifying features typically observed for both systems^24–28^. Most notably, *in vitro* firing events were typically in bursts, whereas *in vivo* firing events were predominantly composed of single spikes (Fig. 1a,b; Supplementary Fig. S1). We also noted greater variability within the recorded features among experimental replicates (firing rate, interspike interval, and reduced single spike activity outside of bursts) in the *in vitro* system (Fig. 1c–e).

### Drug effects on spontaneous single-unit activity

**Figure 2:**
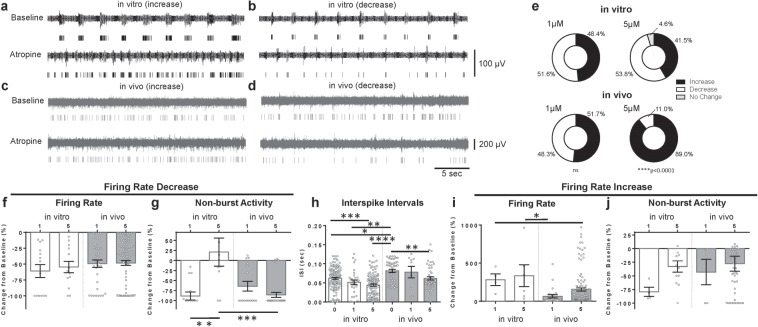
*In vitro* and *in vivo* responses to atropine challenge. Dual response examples for both *in vitro* and *in vivo* systems are shown in (**a**–**d**). Increases and decreases in spiking activity on a single channel when exposed *in vitro* are shown in (**a**,**b**), respectively. *In vivo* responses are shown in (**c**) (increase) and (**d**) (decrease). The proportions of single units showing increase, decrease, or no change in firing activity after exposure are shown in (**e**). A difference in distribution between *in vitro* and *in vivo* single-unit response types for atropine was noted: 1 µM, *Χ*^2^ (2, n = 94) = 1.92, p = 0.38 and 5 µM, *Χ*^2^ (2, n = 354) = 83.77, p < 0.0001. For the cells in (**e**) that showed decreased single unit activity, (**f**) presents the decreases in firing rate *in vitro* (1 µM n = 14; 5 µM n = 13) and *in vivo* (1 µM n = 35; 5 µM n = 90). (**g**) Shows the change in non-burst firing activity for this same group of cells that showed decrease in firing rate to atropine: *in vitro* (1 µM n = 14; 5 µM n = 10), *in vivo* (1 µM n = 16; 5 µM n = 35). (**h**) Shows interspike interval (ISI) for all cells at each dosage of atropine: *in vitro* (0 µM n = 135; 1 µM n = 30; 5 µM n = 112), *in vivo* (0 µM n = 51; 1 µM n = 9; 5 µM n = 54). For the cells in (**e)** that showed increase in cell activity, (**i**) presents the increases in firing rate: *in vitro* (1 µM n = 4; 5 µM n = 6), *in vivo* (1 µM n = 26; 5 µM n = 121). (**j**) Shows the change in non-burst firing activity for this same group of cells that showed decrease in firing rate to atropine: *in vitro* (1 µM n = 4; 5 µM n = 12), *in vivo* (1 µM n = 5; 5 µM n = 45). Data are shown as mean ± s.e.m. Asterisks (*) in panel represent significance from Dunn’s post-hoc test. *p < 0.01, **p < 0.05, ***p < 0.001, ****p < 0.0001.

Upon exposure to atropine, single-unit responses for firing rate and non-burst activity were similar between the systems (Supplementary Fig. S2a,b). Importantly, the expected dual response was observed in both neuronal systems, whereby isolated single units exhibiting simultaneously increasing or decreasing responses to atropine were observed both *in vitro* (Fig. 2a,b) and *in vivo* (Fig. 2c,d) after exposure to 1 µM atropine. This dose of atropine caused no statistically significant differences in response between the two systems, as characterized by both the ratio of cells showing each type of response (Fig. 2e) and by changes in features such as firing rate (Fig. 2f) and interspike interval (Fig. 2h). Responses to the 5 µM dose differed markedly (Fig. 2e) with respect to cells showing a decrease in activity (~10% *in vivo* vs. >60% *in vitro*), although the overall change in firing rate for these cells does not change with dose (Fig. 2f). For both concentrations evaluated, only the 5 µM exposure demonstrated statistically significant differences in the single-spike activity between the two systems (Fig. 2g). Cells *in vitro* appeared more likely to increase in firing rate after atropine exposure, although the response was not statistically significant compared to the *in vivo* response from similar exposures (Fig. 2i). Changes in single-spike activity also showed a similar but statistically insignificant trend (Fig. 2j).

**Figure 3:**
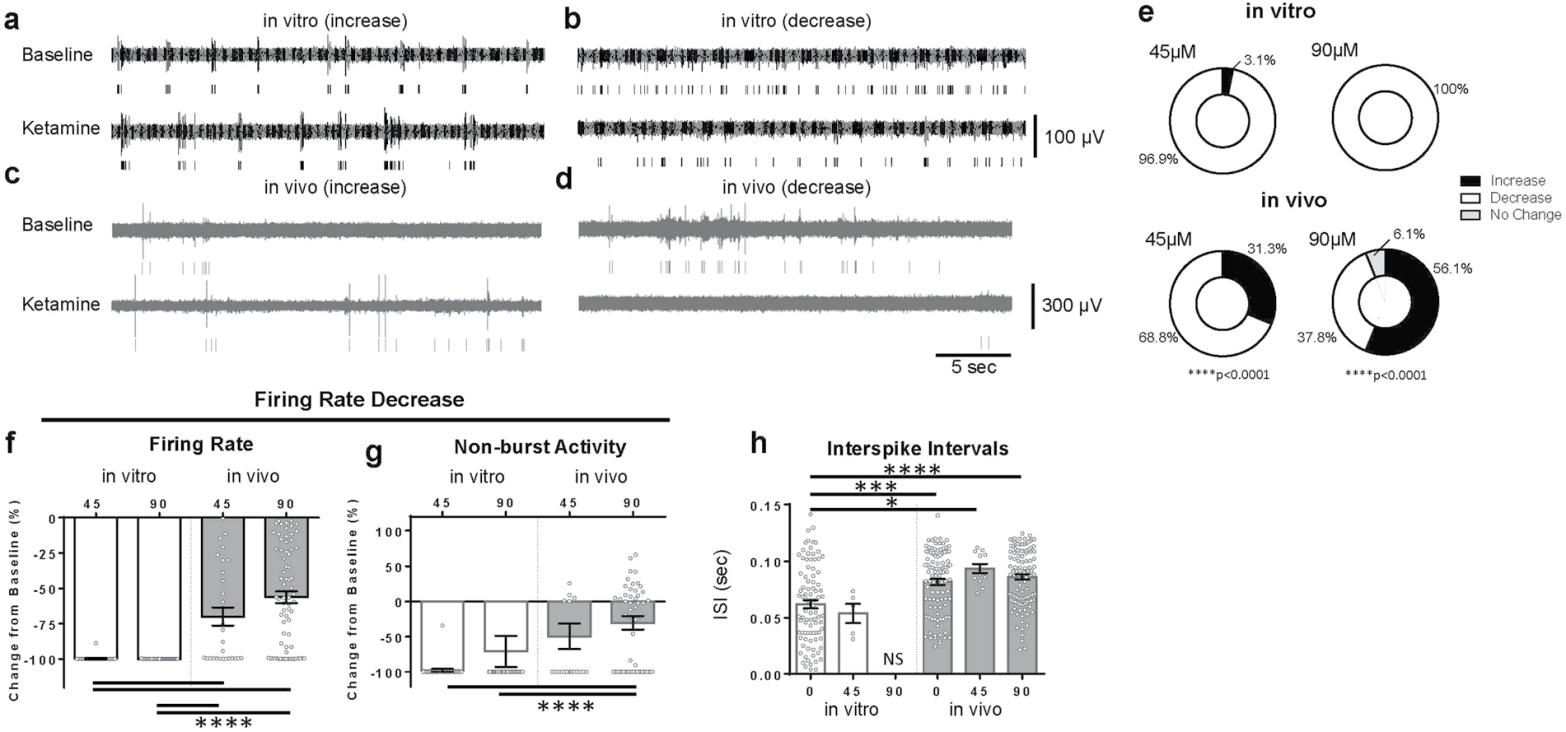
*In vitro* and *in vivo* responses to ketamine challenge. Dual response examples for both *in vitro* and *in vivo* systems are shown in (**a**–**d**). Increases and decreases in spiking activity during simultaneous exposure to 1 µM ketamine *in vitro* are shown in (**a**,**b**), respectively. *In vivo* responses are shown in (**c**) (increase) and (**d**) (decrease) to ~90 µM ketamine. Outcomes after ketamine exposure are shown in (**e**). *In vitro*, 3.1% of cells showed increased activity and 96.9% of cells showed decreased activity to 45 µM ketamine. 100% of cells showed decreased activity to 90 µM ketamine. *In vivo*, 31.2% of cells increased activity and 68.8% of cells decreased activity to 45 µM ketamine. For 90 µM ketamine, 56.1% of cells increased activity, 37.8% of cells decreased activity, and 6.1% of cells showed no response. A difference in distribution between *in vitro* and *in vivo* single-unit response types for ketamine was noted: 45 µM, *Χ*^2^ (2, n = 85) = 17.08, p < 0.0001, and 90 µM *Χ*^2^ (2, n = 229) = 43.96, p < 0.0001. For the cells in (**e)** that showed decreased single unit activity, (**f**) presents the decreases in firing rate: *in vitro* (45 µM n = 31; 90 µM n = 33), *in vivo* (45 µM n = 29; 90 µM n = 74), (**g**) presents the decreases in non-burst activity: *in vitro* (45 µM n = 31; 90 µM n = 33), *in vivo* (45 µM n = 21; 90 µM n = 59), and (**h**) presents the interspike intervals (ISI): *in vitro* (0 µM n = 90; 45 µM n = 5; 90 µM n = 1), *in vivo* (0 µM n = 100; 45 µM n = 12; 90 µM n = 109). Response to vehicle is reported as 0 µM. Data are shown as mean ± s.e.m. Asterisks (*) in panel represent significance from Dunn’s post-hoc test. *p < 0.01, ***p < 0.001, ****p < 0.0001.

Next, we investigated the effects of ketamine in both systems. Studies have reported that at sub-anesthetic doses *in vivo*, ketamine induces both increases and decreases in cortical neuron firing rate. At higher, anesthetic doses, most neurons exhibit a reduced firing rate, while a small but significant subset of neurons still exhibits a firing rate increase^20^. In the current study, we observed ketamine’s dual response behavior both *in vitro* and *in vivo* (Fig. 3). Overall, cortical neurons *in vitro* demonstrated greater sensitivity to ketamine exposure, with approximately one order of magnitude decrease in the dose range *in vitro* required to elicit the same response as *in vivo* (Supplementary Fig. S3). The difference in sensitivity is further illustrated in Fig. 3e and Supplementary Fig. S2c,d, which examines single-unit responses for the two concentrations of ketamine (45, 90 µM) that elicited responses in both systems. Nearly all cells *in vitro* (>99%) showed a decrease in activity when exposed to either 45 or 90 µM ketamine. *In vitro*, this decrease is reflected as a near complete cessation of activity (Fig. 3f); this was not the case for ≤1 µm ketamine (Supplementary Fig. S3) where a subset of *in vitro* cells showed increased activity. *In vivo*, however, fewer cells demonstrated decreases in activity for both 45 µM and 90 µM ketamine (68.8% and 37.8%, respectively). This reduction corresponded to a 40–50% decrease in activity for these cells (Fig. 3f). Interspike intervals (ISI) for both systems did not show a significant change from baseline (0 µM ketamine, Fig. 3h), aside from the *in vitro* exposure to 90 µM ketamine, which showed a complete (but reversible, Supplementary Fig. S4) cessation in single-unit activity. Finally, Fig. 3g shows a trend towards less non-burst firing *in vitro* compared to *in vivo*, similar to the changes in firing rate seen in Fig. 3f. This does not indicate an increase in bursting, but instead indicates decreases in single spike activity overall when interpreted in the context of the firing rate decreases in Fig. 3f.

## Discussion

This study aimed to quantify the similarities and differences of cultured cortical neurons and cortical neurons *in vivo* by comparing spontaneous electrophysiological activity and changes in electrophysiology after exposure to atropine or ketamine. These experiments showed: (1) significant differences in spontaneous *in vivo* and *in vitro* firing activity, (2) similar response trends in both systems in single-unit spiking activity after exposure to atropine, (3) greater sensitivity to the effects of ketamine in neurons *in vitro*. It is important to be aware that several factors could influence the differences observed in this study. These factors include: the age of neurons (embryonic versus adult)^29^, the ratio of GABA to glutamatergic cells^15^, presence and composition of supporting glial cells^30^, and the isolated environment for the *in vitro* system which lacks input from other organs^31,32^. Each of these points will be discussed in greater detail below.

*In vitro* cultures typically rely on embryonic neurons, as cell viability after harvesting and long-term culturing is more robust in embryonic and postnatal rodent systems^33,34^ compared to adult^35–37^. Electrical properties for neurons cultured *in vitro* at different ages have been compared and found to be similar across age groups^29^ apart from bursting behavior, which was more common in embryonic than adult neurons^29^. This characteristic of embryonic neurons may contribute to the differences observed here for spontaneous baseline neuronal activity between the two systems. Cultured neurons demonstrated more burst-like features (i.e. fewer spikes out of bursts) compared to neurons *in vivo*. With these inherent differences between systems in mind, responses to chemical stimuli were evaluated relative to the spontaneous (baseline) activity of the given system. This evaluation allowed for a more direct comparison for responses between systems.

The composition and ratios of neuronal subspecies, as well as the level of their direct interaction and feedback ability, are considerably different between *in vivo* and *in vitro* systems. Previous work has shown that a NMDA antagonist similar to ketamine, MK-801, decreases the neuronal activity of pyramidal cortical cells in culture and anesthetized rats^38^ while increasing cell activity in pyramidal cortical cells in awake rats^39^. This difference was attributed to decreased GABAergic interneuron activity in the awake animal, resulting in disinhibition of cortical neurons and a subsequent increase in spiking activity^27^. In cultured neurons or in anesthetized neurons *in vivo*, this disinhibition of cortical neurons is ineffective due to low basal levels of extracellular glutamate, the additional factor required to induce pyramidal cell action potentials^40,41^. Additionally, rodent cortical neurons in culture show an increased sensitivity to the excitotoxic effects of glutamate^42^ and neuronal NMDA receptors show differing sensitivity depending on the developmental stage of the neurons, with a hypersensitive stage seen in the first two weeks of rat neonatal life^43^. These factors, as well as the differences in GABAergic neurons between systems, may have contributed to the differential responses observed for ketamine. Within our *in vitro* system a small percentage (~8%, Supplementary Fig. S5) of neurons are GABAergic, compared to ~20% *in vivo*^15^. Supporting glia composition also differed between systems. Neuroglia including astrocytes, oligodendrocytes, and microglia all play a role in maintaining and influencing neuronal health and function^44,45^. While our *in vitro* system contains some contaminating astrocytes (GFAP^+^, ~13%, Supplementary Fig. S5), biologically relevant ratios and other cell types are not present. Cortical regions *in vivo* contain a much larger percentage of glial support cells (~40%)^46^ which are known to help protect against deleterious glutamate effects and possess NMDA receptors themselves^47^.

While differences in composition and ratios of cell types between systems may have influenced the response to ketamine, atropine responses were more comparable. Expression of muscarinic receptors is low in the embryonic rat cerebral cortex (E18; 5% of adult levels) but receptor expression increases rapidly through the first 11 days postnatally^30^ before stabilizing at levels that will be maintained through adulthood^48^. Interestingly, Heacock *et al*. showed that despite low expression of muscarinic receptors in embryonic neurons, their sensitivity to the agonist carbachol was similar to that of adult neurons^30^. The authors attributed the comparable sensitivity to a change in effective turnover rate (or the addition of unused receptors to cerebral cortical neurons as rats age) to explain why the neurons respond similarly to muscarinic agonists despite the differences in the maturity of the cultured neurons. A similar argument could explain why our *in vivo* and *in vitro* systems responded similarly to atropine. Additionally, muscarinic receptor expression on supporting cell types such as microglia and oligodendrocytes does not change with age in the rodent model^49,50^. Therefore, the recognized differences in ratios of neuronal to supporting cells for both systems may have not affected overall response.

As isolated tissue mimetics, *in vitro* model systems lack the ability to metabolize drug compounds. Therefore, the metabolites found after drug administration *in vivo*, and their potential off-target effects, are likely not recapitulated *in vitro*. This represents a significant difference between the two systems, particularly the differing responses seen for ketamine, a drug known to be readily metabolized *in vivo*. Importantly, ketamine metabolites have known neuroactive properties, and can act on other receptors to counter ketamine effects^21^ or directly work on the same receptors as ketamine with less efficacy^31^. These neuroactive metabolites may have contributed to the differences observed between our experimental systems.

Neuromodulatory inputs from other regions are absent in the *in vitro* system; these inputs influence cortical neurons *in vivo*^32^. The cortex is ~25% of the rodent brain’s total volume^51^ but is comprised of many specialized subregions that are a result of these interregional inputs. The input for each individual cell in the cortex is influenced by the larger circuitry of the brain; this can also have additional, complex effects on the ultimate response a single neuron shows to systemically applied drugs.

Taken together, our results demonstrate that there are quantifiable differences and similarities in extracellular electrophysiology between *in vivo* and *in vitro* cortical systems both for spontaneous activity and in response to chemical challenge with atropine and ketamine. This study represents only a preliminary step towards a more thorough comparison between *in vivo* and *in vitro* responses, which would require testing additional representative drug compounds across multiple drug classification categories. These more extensive comparisons are necessary to correlate and benchmark the responses between the two systems to better interpret *in vitro* data in the context of the whole organism. While the complexity of the *in vivo* system is a key determinant of chemical response, directly demonstrating the ability of the relatively simple *in vitro* system to recapitulate the fundamental response observed *in vivo* is critically important. Using two different classes of drugs, we demonstrate the utility of cortical *in vitro* neuronal systems to recapitulate whole organ responses to certain classes of chemicals. While we show that changes in extracellular electrical activity in neuronal cultures may be appropriate for certain types of pharmacological studies, we also demonstrate that this system may not fully recapitulate the responses observed *in vivo*. Understanding the boundaries of a representative response with this *in vitro* system will provide more relevant data sets, and a framework to develop *in vitro* systems that more closely mimic *in vivo* function and response. As such, in addition to expanding the number and type of drug compounds to test, future work should be aimed at developing more complex cultures (i.e. additional cell types, three dimensional cultures) that capture more of the whole brain response for complex drugs of action like ketamine, while concomitantly verifying that functional responses pivot towards a more *in vivo* functional phenotype. However, until more complex cultures or alternative *in vitro* systems have been developed and validated, we hope to begin a larger conversation between *in vitro* and *in vivo* communities to understand the right questions we can correctly answer with *in* *vitro* platforms to directly contribute to human health and disease.

## Methods

### Chemicals

All chemicals were purchased from Sigma-Aldrich (St. Louis, MO) unless otherwise noted. Ketamine HCl (100 mg/mL in sterile water) was purchased from ACE surgical (Brockton, MA).

### Microelectrode device fabrication

The MEA device used for *in vitro* platform testing was microfabricated as previously described^52,53^. Briefly, MEAs (20–50 µm platinum electrodes spread evenly over 1.8 mm^2^) were fabricated on a glass substrate. Standard photolithography and wet or plasma etching were used to pattern metal constituting the 60 electrodes and electrode traces; polyimide was deposited and patterned as an insulating layer over the metal traces. A polystyrene cylinder was affixed over the MEA to enable cell culturing over the array. The well area was 113 mm^2^ and accommodated approximately 700 μL. ZIF connectors were added to the device for electrical connections and electrodes were plated with platinum black^53^. Impedance measurements were taken prior to seeding and ranged from ~50–250 kΩ at 1 kHz.

The MEA devices implanted *in vivo* were microfabricated and plated as described previously^54,55^. Briefly, the implantable MEAs featured two separate shanks (each 15 μm thick and 100 µm wide) spaced 2 mm apart medial-laterally. At the tip of each shank, 16 electrodes were evenly distributed over a length of 1.89 mm (dorsal-ventrally through medial prefrontal cortex). MEA devices were fabricated on silicon substrates using alternating stacks of metal (conductive layers) and polyimide (insulation layers). Standard photolithography and wet or plasma etching were used to pattern the 32 electrodes, trace metal, and device outlines. Polyimide was deposited and patterned, followed by deposition of platinum for the electrode metal. The electrode metal area was then patterned and etched creating electrodes 20 μm in diameter, followed by an insulating polyimide layer. Next, layers of gold (for the metal traces connecting the electrodes to the connector region) and polyimide were deposited to create a layered structure. After fabrication, devices were released from the silicon wafer and individually connected to 36-channel Nano Strip connectors (Omnetics Connector Corporation, Minneapolis, MN) using conductive epoxy. Electrodes were then plated as described above with final impedances ranging from 50–400 kΩ at 1 kHz and prepped for surgical implantation.

### Cell seeding

MEAs were sterilized with 70% ethanol for 20 minutes, rinsed 5x with sterile water, and coated with 0.1 mg/mL poly-D-lysine in borate buffer prior to seeding. Primary embryonic rat cortical neurons (E18/19, Lonza, Walkersville, MD) were seeded at a density of 180 K cells/device (N = 4 seedings, n = 16 devices, [lot #170505 and #091104]). Cultures were maintained with bi-weekly exchanges of 50% media (Primary Neuron Basal Medium (PNBM) supplemented with 2 mM L-glutamine, 50 μg/mL gentamicin, 37 ng/mL amphotericin, and 2% NSF-1) in a humidified incubator (5% CO_2_, 37 °C). Custom device caps, made from a polytetrafluoroethylene (PTFE) housing and a fluorinated ethylene-propylene (FEP) membrane, were used to maintain sterility and to allow for gas exchange.

### *In Vivo* Surgical Implantation

All animal experiments and surgical procedures were conducted following the guidelines and regulations set by Lawrence Livermore National Laboratory, including Institutional Animal Care and Use Committee approval (#240). Devices were surgically implanted into medial Prefrontal Cortex (2.52 mm anterior-posterior, 1.2–1.8 mm medial-lateral, 1.5 mm dorsal-ventral) of male Sprague-Dawley rats (6–8 weeks, ~200 g). The device, with a silica insertion tool temporarily attached to the probe with polyethylene glycol (PEG), was implanted using a microdrive positioner (Kopf Instruments, Tujunga, CA). The PEG holding the positioner was then dissolved with saline and the insertion tool was retracted with the microdrive, leaving the array implanted in the brain^54,56^. The array connector was then affixed to the skull with dental acrylic.

### Chemical dosing

For *in vitro* administration, chemicals were either dissolved (atropine; powder, >99.9% purity) or diluted (ketamine) in primary neuron basal media (PNBM) to 4X the final desired *in vitro* concentration. Cultures used for chemical testing were between day *in vitro* (DIV) 19 and 78. For chemical exposures, 25% of the culture media was removed from the MEA well and replaced with an equal volume of 4X chemical working stock (0.0004–360 µM ketamine, 4–10 µM atropine), resulting in a final culture concentration that ranges from 0.0001–90 µM for ketamine and 1–5 µM for atropine.

For *in vivo* administration, chemicals were either dissolved (atropine powder) or diluted (ketamine solution) in 0.2–0.4 mL saline (0.9% NaCl in sterile water). Intraperitoneal (i.p) dosing concentrations were based on published studies that evaluated brain region specific accumulation of non-metabolized (radiolabeled) drug upon i.p. injection^57,58^. Atropine (10 mg/kg) was given i.p. to rats (400–550 g); this was equivalent to 1 µM atropine in cortex (and 50 mg/kg = 5.2 µM). For ketamine, a concentration of 45 µM in the cortex was achieved with 5 mg/kg i.p. administration (and 10 mg/kg i.p. for 90 µM, 25 mg/kg for 200 µM).

### Electrophysiology recording and processing

Parallel *in vivo* and *in vitro* experiments began on the day of probe implant *in vivo* (Day *In Vivo*) or day of cell seeding on the MEAs (Day *In Vitro*). Time from probe implant or cell seeding were both referred to as DIV, with DIV 0 referring to the day of *in vivo* probe implantation or *in vitro* cell seeding. Electrophysiology recordings began as early as DIV 3 to acclimate the animal to the recording chamber and to monitor the health of the *in vitro* cultures. Chemical challenges were initiated on DIV 21, when neuroinflammation from the surgery was resolved *in vivo*^54^ and the cultures began to show stable neuronal activity *in vitro*^24^.

For *in vitro* electrophysiology measurements, devices were placed on a heated stage (37 °C) in a 5% CO_2_ controlled chamber during recordings. Electrophysiology measurements were acquired using a multi-channel recording system (AlphaLab SnR, Alpha Omega, Alpharetta, GA) and were sampled at a frequency of 22.3 kHz and bandpass filtered between 268 and 8036 Hz. Baseline and chemical exposure measurements were recorded for 20 minutes. Chemical exposure measurements were collected for 20 minutes immediately following chemical addition. After testing, cultures were given at least two days for recovery between experiments.

For *in vivo* measurements, drug application was randomized so that there were no more than two recordings from the same animal in the same week. Each rat (N = 3) was administered both concentrations of each drug three times over a period of 6–12 weeks with at least two days between experimental drug exposures. For each drug administration, animals were placed in an operant behavioral chamber (Med Associates, Inc., St. Albans, VT) and acclimated to the chamber for >15 minutes before recording was initiated. Electrophysiology was recorded using a multichannel freely moving animal system (Tucker-Davis Technologies, Alachua, FL) at 24,414.1 Hz with a band pass filter of 100–5000 Hz. Baseline was monitored to ensure proper connectivity of devices and noise thresholds (auto set to ±4.0 standard deviations above noise). Animals then were administered a vehicle injection of 0.2–0.4 mL saline (0.9% NaCl in sterile water, i.p.) and 20 min of baseline condition data was collected. Next, animals were injected with the chemical challenge dissolved in 0.2–0.4 mL saline (0.9% NaCl in sterile water, i.p.) and >20 min of chemical condition data were then collected.

### Feature analysis and statistics

After collection, all *in vivo* and *in vitro* data were analyzed to extract single-units from the multiunit signal data using Offline Sorter™ (PLEXON, Dallas, TX) and further analysis carried out only on isolated single-units. For all isolated single units, spike and burst features were calculated. Each single unit was analyzed individually and considered a data point for the experiment since each cell could respond differently to both drugs; averaging responses across a device would confound results and interpretation. Electrophysiological data from each unit on the *in vitro* MEA device or *in vivo* probe was analyzed using parameters we have previously evaluated on cortical and hippocampal cultures seeded on MEA devices^53^. Bursts were defined as having a maximum beginning interspike interval of 0.1 sec, a maximum end interspike interval of 0.2 sec, a minimum interburst interval of 0.5 sec, a minimum burst duration of 0.05 sec, and a minimum of 10 spikes per burst^53^. Feature analysis was carried out with an in-house custom R package.

Calculated features were inspired by those described in the work of Charlesworth *et al*.^24^: interspike interval (ISI), overall firing rate and percent of spikes outside of bursts. For drug challenges, the difference in electrophysiological activity before and after challenge was calculated and reported as the change in mean frequency from baseline. Here, n indicates the number of isolated single units. Single units that showed a complete cessation of firing activity with drug challenge were defined at a -100% change in activity. Single units exhibiting greater than 1000% change in the feature were excluded. Data was considered non-normally distributed for statistical analysis and were analyzed using GraphPad Prism7. *In vivo* and *in vitro* baseline firing were compared with the non-parametric Mann-Whitney test (Fig. 1c–e). For drug challenges, a chi-square test was run on the proportions of single units showing an increase, decrease, or no change for both systems (Figs 2e and 3e). Differences between multiple groups for features (Figs 2f–j and 3f–h, Supplementary Figs S2 and S3) were evaluated by the non-parametric Kruskal-Wallis one-way analysis of variance (ANOVA), followed by Dunn’s *post hoc* test. A p value of <0.05 was considered significant. All error bars indicate standard error of the mean (SEM).

### Code availability

The analysis code used to analyze data in this study is available upon request.

### Immunocytochemistry

Cells were rinsed 4X with 1X PBS, fixed with 4% paraformaldehyde, washed with PBS (4X), and permeabilized using 10% saponin before blocking with 10% goat serum (1 hr at room temperature). Primary antibodies commonly used to identify cell populations were used: class III beta-tubulin for neurons (Tuj-1, chicken or mouse, Neuromics, Edina, MN, 1:200 dilution), GAD-67 for GABAergic neuron (mouse, Millipore, Burlington, MA, 1:200) and glial fibrillary acidic protein (GFAP) for astrocytes (rabbit, Millipore, Burlington, MA, 1:1000). After primary antibody incubation (overnight at 4 °C), cells were washed with PBS (4X) and stained with secondary antibodies (1 hr at 37 °C). Secondary antibodies included: goat anti-mouse linked to Alexa Fluor 488, goat anti-chicken linked to Alexa Fluor 647 and goat anti-rabbit linked to Alexa Fluor 594 (1:500 dilution, Life Technologies, Eugene, OR). After secondary antibody incubation, the cells were washed with PBS (4X), and incubated (20 min) with the nuclear stain, diamidino-2-phenylindole (DAPI, ThermoFisher, 300 nM), before imaging. Three fields of view (or regions) from each cell culture were imaged and acquired using a Leica inverted microscope controlled using Meta-morph imaging software (Molecular Devices, Sunnyvale, CA) for Supplemental Fig. 1d–f. A LSM700 confocal microscope (Carl Zeiss Microscopy, Thornwood, NY) was used for Supplemental Fig. 1a–c. For cell type quantification, positive cells (GFAP^+^, astrocyte marker and GAD-67^+^, GABAergic neuron marker) were counted and averaged from three to five fields of view of each culture (n = 3) and normalized to the average total nuclei count or Tuj-1 positive cells using ImageJ.

### Histology

*In vivo* probe placement was verified after CO_2_ euthanasia of animals. Rats were perfused using a 10% formalin solution. Brains were removed, cryoprotected, and stored in 10% formalin for >3 days. Tissue was then sectioned coronally into 40–50 μm thick slices on a cryostat (Leica CM3050 S, Buffalo Grove, IL). The probe tract was identified visually under a microscope equipped with a camera. Approximate recording sites are shown in Supplementary Fig. 6.

### Data availability

The data that support the findings of this study are available from the corresponding authors upon reasonable request.

## Electronic supplementary material

Supplementary Figures
