# Supplementary material for: Evaluation of in vitro neuronal platforms as surrogates for in vivo whole brain systems
Source: Sci Rep. 2018 Jul 17;8:10820. doi: 10.1038/s41598-018-28950-5 (PMC6050270; doi:10.1038/s41598-018-28950-5)
Supplement: Supplementary file 1 — Supplementary Figures [file 41598_2018_28950_MOESM1_ESM.pdf]

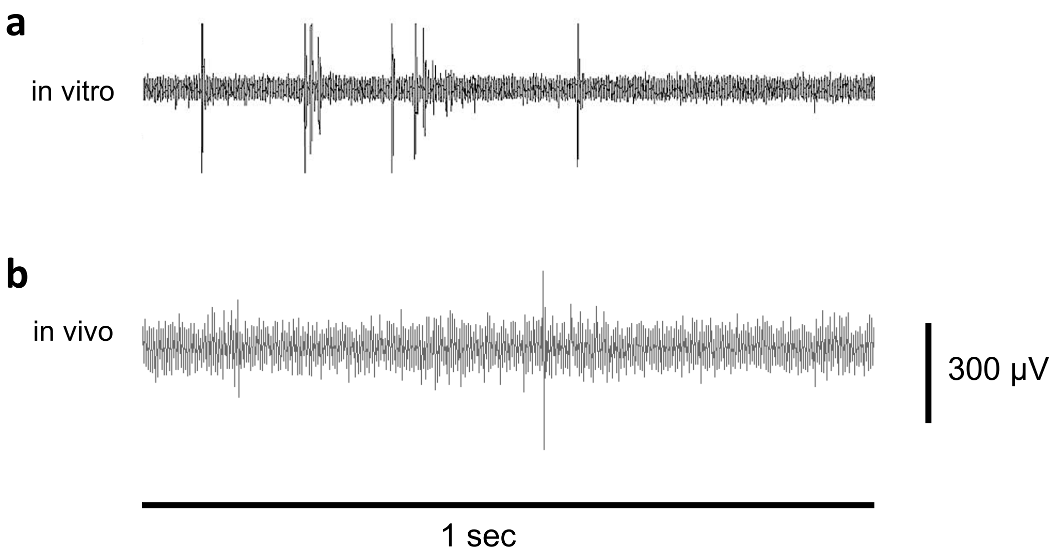
a
in vitro
b
in vivo
300 μV
1 sec

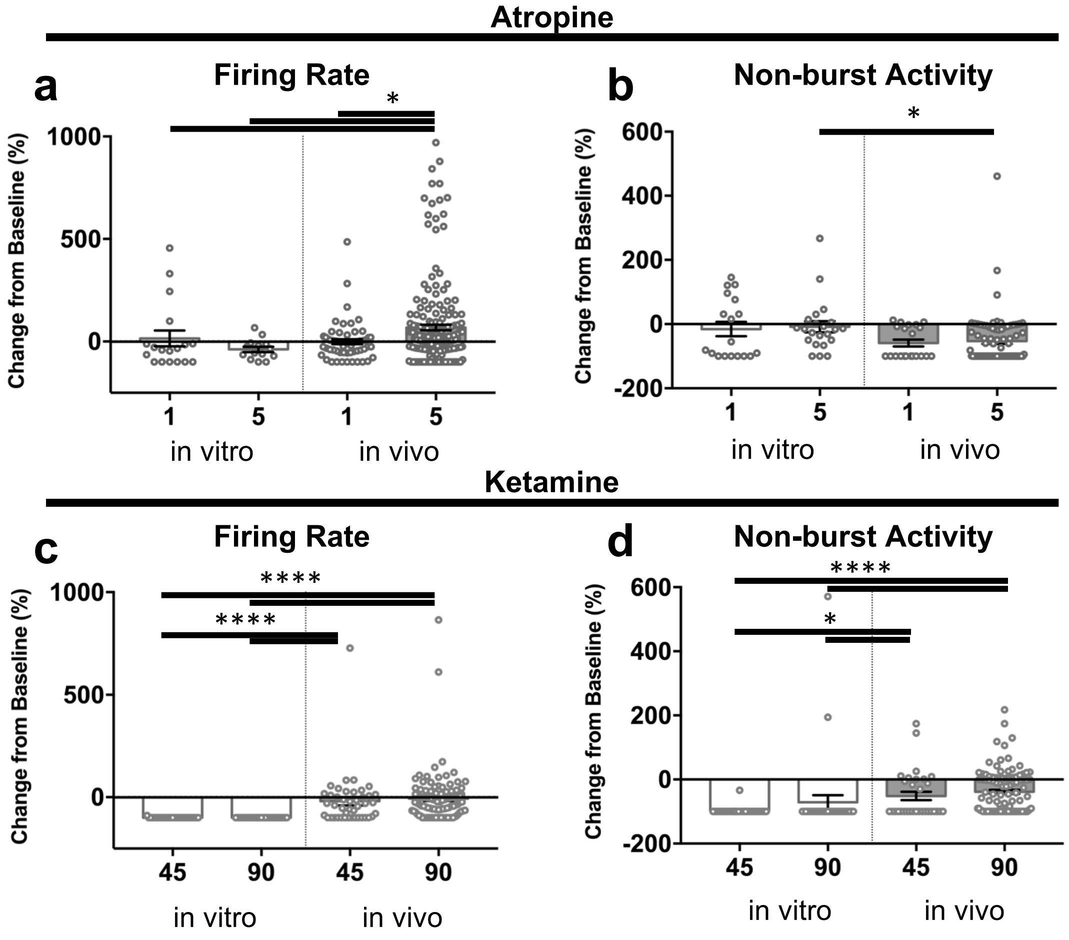
Atropine
a
Firing Rate
*
Change from Baseline (%)
1000
500
0
1
5
1
5
in vitro
in vivo
b
Non-burst Activity
*
600
400
200
0
-200
Ketamine
c
Firing Rate
****
****
45
90
45
90
d
Non-burst Activity
****
*

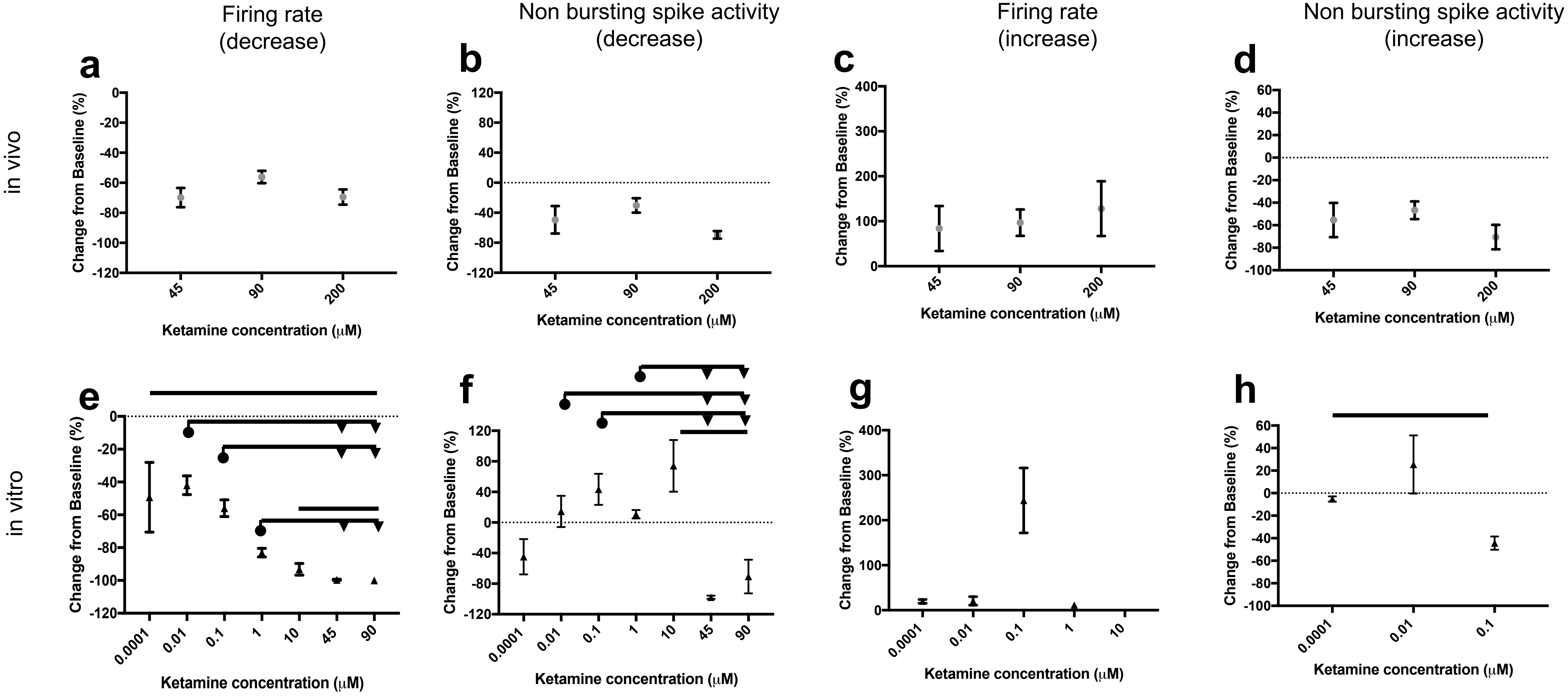
Firing rate
(decrease)
Non bursting spike activity
(decrease)
Firing rate
(increase)
Non bursting spike activity
(increase)
in vivo
in vitro
a
b
c
d
e
f
g
h
Change from Baseline (%)
Ketamine concentration (μM)

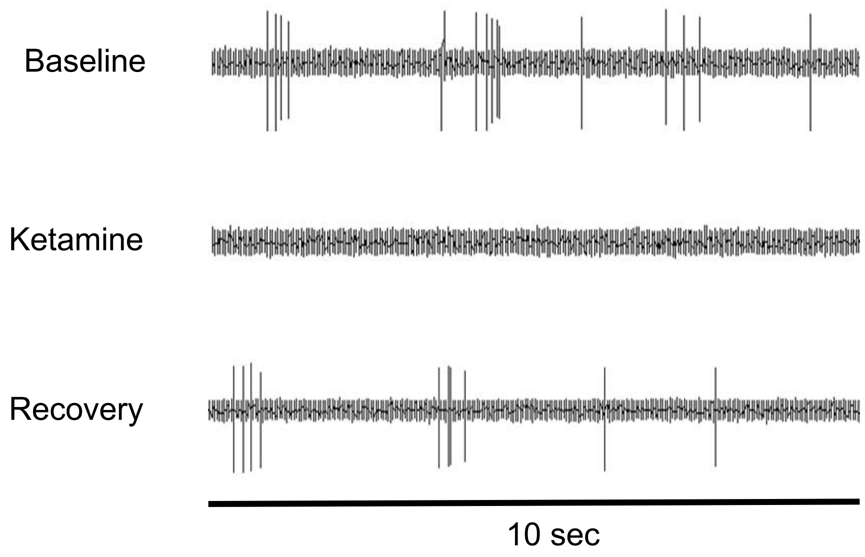
Baseline
Ketamine
Recovery
10 sec

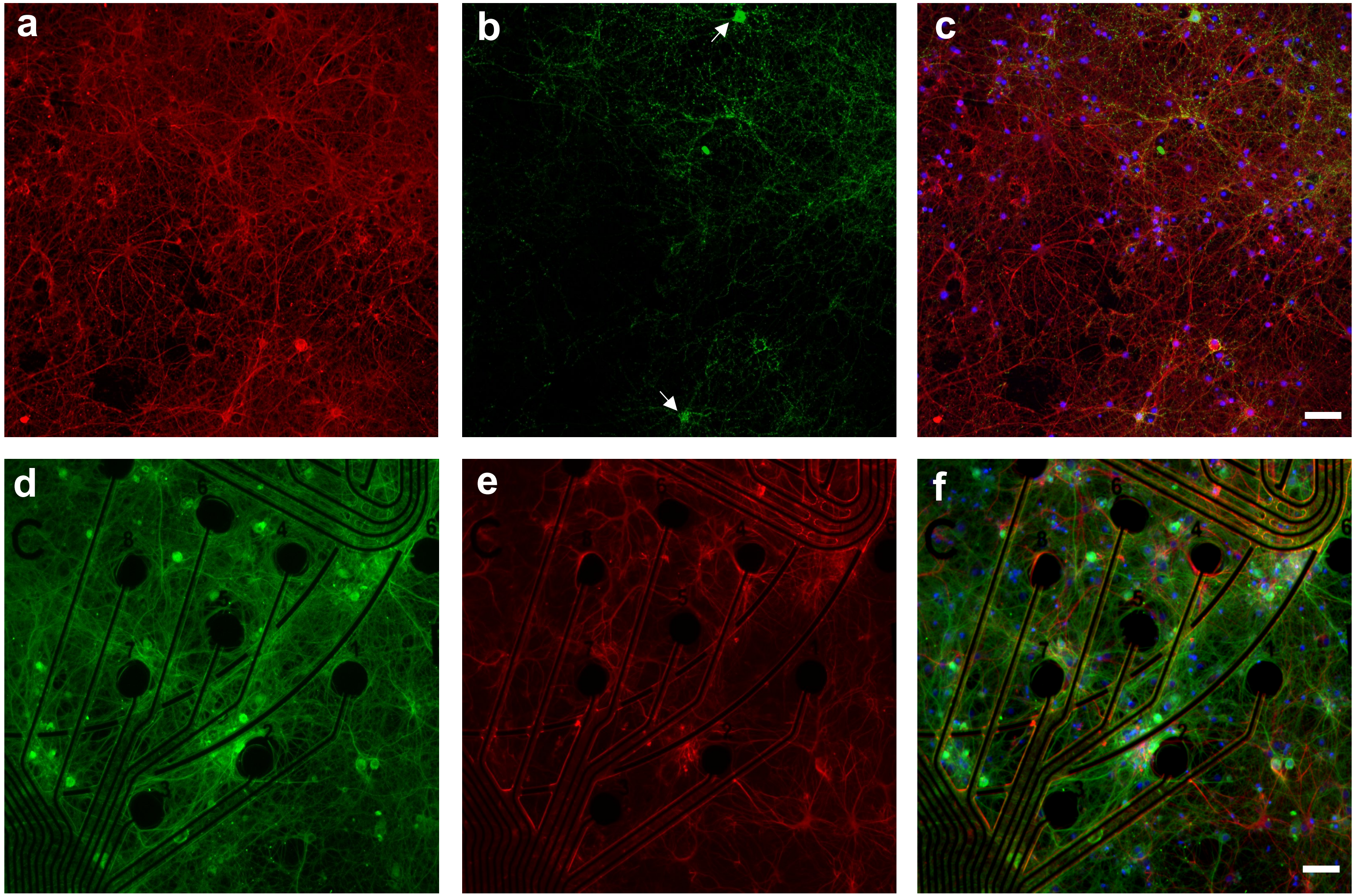
a
b
c
d
e
f

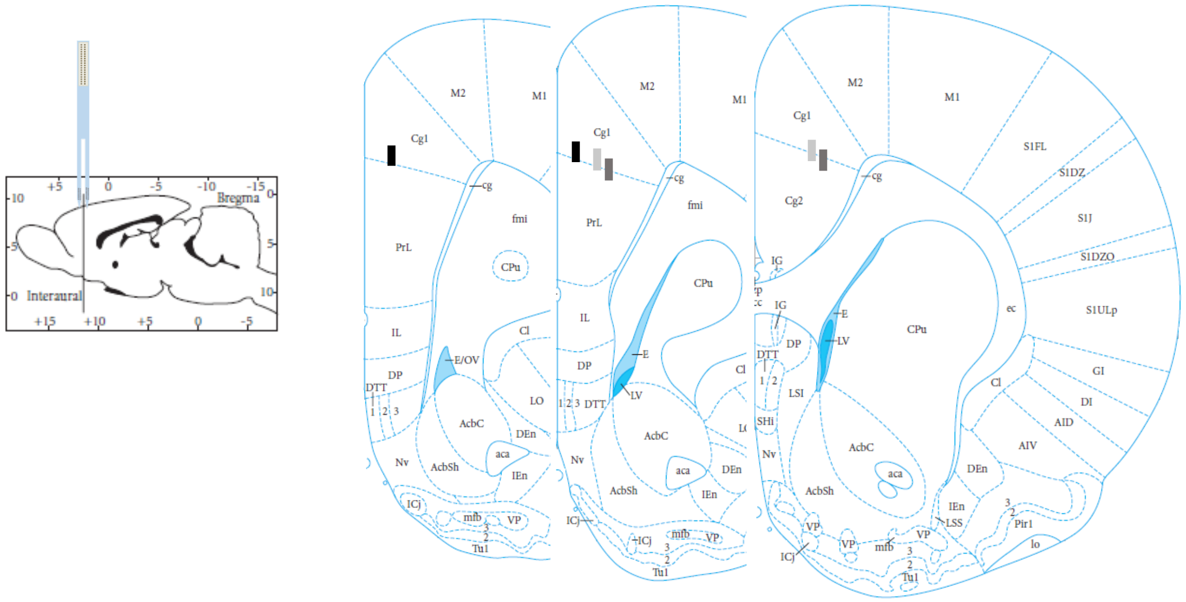

Bregma +2.76 Bregma +2.52 Bregma +2.28

Supplementary Table 1. Summary of responses for in vivo cells that show decreased firing activity to ketamine. (SI Fig S3 a &b)

Dosage Ketamine	Average decrease firing rate ± SEM (%)	Number of cells	Average change nonburst spikes ± SEM (%)	Number of cells
45 µM	-69.82 ± 6.41	29	-49.5 ± 18.3	21
90 µM	-56.14 ± 4.07	74	-30.3 ± 9.6	59
200 µM	-69.5 ± 5.06	33	-61.4 ± 10.3	21

Supplementary Table 2. Summary of responses for in vivo cells that show increased firing activity to ketamine. (SI Fig S3 c & d)

Dosage Ketamine	Average increase firing rate ± SEM (%)	Number of cells	Average change nonburst spikes ± SEM (%)	Number of cells
45 µM	84.0 ± 50	14	-55.4 ± 15.2	10
90 µM	96.8 ± 29.4	34	-46.8 ± 7.8	50
200 µM	127.9 ± 60.8	10	-70.6 ± 10.8	9

Supplementary Table 3. Summary of responses for in vivo cells that show decreased firing activity to ketamine. (SI Fig S3 e & f)

Dosage Ketamine	Average decrease firing rate ± SEM (%)	Number of cells	Average change nonburst spikes ± SEM (%)	Number of cells
0.0001 µM	-49.3 ± 21.2	5	-44.8 ± 23.1	5
0.01 µM	-42.0 ± 5.7	25	14.5 ± 20.5	13
0.1 µM	-55.9 ± 5.1	24	43.4 ± 20.2	17
1.0 µM	-83.1 ± 2.6	16	10.8 ± 5.6	10
10 µM	-93.2 ± 3.6	11	74.1 ± 33.9	8
45 µM	-99.63 ± 0.4	31	-97.9 ± 2.1	31
90 µM	-99.98 ± 0.01	33	-70.7 ± 22.0	33

Supplementary Table 4. Summary of responses for in vivo cells that show increased firing activity to ketamine. (SI Fig S3 g & h)

Dosage Ketamine	Average decrease firing rate ± SEM (%)	Number of cells	Average change nonburst spikes ± SEM (%)	Number of cells
0.0001 µM	19.8 ± 4.1	8	-5.2 ± 2.3	8
0.01 µM	20.6 ± 9.5	5	25.4 ± 25.8	2
0.1 µM	243.9 ± 72.1	13	-44.3 ± 5.8	35
1.0 µM	11.6 ± 0	1	n/a	0
10 µM	n/a	0	n/a	0
45 µM	n/a	1	n/a	1
90 µM	n/a	0	n/a	0

Evaluation of in vitro neuronal platforms as surrogates for in vivo whole brain systems

Anna M. Belle[1,3], Heather A. Enright[2,3], Ana Paula Sales[1], Kristen Kulp[2], Joanne Osburn[2], Edward A. Kuhn[2], Nicholas O. Fischer[2]*, Elizabeth Wheeler[1]*

[1]Engineering Directorate, Lawrence Livermore National Laboratory, Livermore, California, USA

[2]Physical and Life Science Directorate, Lawrence Livermore National Laboratory, Livermore, California, USA

[3]These authors contributed equally

*email: fischer29@llnl.gov; wheeler16@llnl.gov

Supplementary Figure S1. Representative baseline activity of neurons recorded from in vivo and in vitro multielectrode arrays (MEAs). Neuronal activity over one second are shown for in vitro and in vivo recordings in **a** and **b**, respectively.

Supplementary Figure S2. In vitro and in vivo responses to atropine and ketamine challenges. Reponses include single units showing an increase, decrease or no change after challenge. Changes in firing rate are shown for both systems in **a** and **c**. Changes in non-burst firing activity are shown in **b** and **d**. Asterisks (*) in panel represent significance from Dunn's post-hoc test. *p < 0.01, ****p<0.0001.

Supplementary Figure S3. Dose-response to ketamine concentrations tested in vivo (**a-d**) and in vitro (**e-h**). In vivo, for cells that showed a decrease in firing rate to a given dosage of ketamine, **a** presents the average firing rate decrease for these cells, and **b** presents the change in spikes outside of bursts. There is a clear decrease in firing rate with concentration observed in **a.** As dose increases, we see an increase in single spike activity (**b**) until activity is greatly reduced at 45 and 90 µM (see panel **a** with 100% decrease in firing rate). In vivo, for cells that showed an increase in firing rate for a given

dosage of ketamine, **c** presents the average increase in firing rate activity compared to baseline, and **d** presents the change in spikes outside of burst. Firing rate increases (**c**) showed no clear trend with concentration. No trend was observed for spikes outside of bursts for cells that demonstrated an overall firing rate increase (**d**).

In vitro, for cells that showed a decrease in firing rate to a given dosage of ketamine **e** presents the average firing rate decrease for these cells, and **f** presents the change in spikes outside of bursts. For in vitro cells that showed an increase in firing rate for a given dosage for ketamine **g** presents the average increase in firing rate activity compared to baseline and **h** presents the change in spikes outside of burst. Doses missing from graphs in **g** and **h** had no population of cells show activity (0/11, 1/31, 0/33 isolated units for 10, 45 and 90 µM doses, respectively). Ball and arrow bars show where multiple conditions are significantly different from a single condition, whereas solid bars show significant differences between two conditions ($p<0.05$) from Dunn's post-hoc test. Values and n's are summarized in Supplementary Tables S1-4. Data are shown as mean ± s.e.m.

Supplementary Figure S4. Reversibility of drug effect in cell culture. Top panel shows spike traces from a representative 10 seconds of a 20-minute baseline recording from single electrode on 60 electrode MEA in cortical cell culture. Middle panel shows representative 10 seconds of a 20-minute acute exposure to 1 µM ketamine from same electrode on MEA. Bottom panel shows representative 10 seconds of a 20 minutes from recovery period after rinsing ketamine and replacing with standard cell culture media on same MEA electrode.

Supplementary Figure S5. Immunofluorescence characterization of cortical cultures in vitro. **a-c**, Neuronal characterization. **a**, Neurons were identified by staining for Tuj-1

(Neuron-specific class III beta-tubulin). **b,** GABAergic neurons were identified by staining for glutamic acid decarboxylase (GAD67) and were characterized by soma staining (white arrows) **c,** Merged tuj-1 (red) and GAD67 (green) image with nuclear stain (DAPI, blue). **d-f**, immunostaining of neurons and astrocytes on devices in vitro. **d,** Tuj-1 neuronal marker, **e,** GFAP astrocyte staining, **f,** merged tuj-1 (green) and GFAP (red) image with nuclear stain (DAPI, blue).

Supplementary Figure S6. In vivo probe placement. Left inset shows positioning of 2 shanks of probe facing midline. On right, coronal sections ranging from 2.76 to 2.28 anterior to bregma, adapted from Paxinos and Watson (2007). Approximate recording regions for both shanks of each probe are marked in the same color on the sections.

Supplementary Table S1. Summary of responses for in vivo cells that show decreased firing activity to ketamine. (SI Fig 3 a &b)

Supplementary Table S2. Summary of responses for in vivo cells that show increased firing activity to ketamine. (SI Fig 3 c & d)

Supplementary Table S3. Summary of responses for in vivo cells that show decreased firing activity to ketamine. (SI Fig 3 e & f)

Supplementary Table S4. Summary of responses for in vivo cells that show increased firing activity to ketamine. (SI Fig 3 g & h)
